# Stereotactic radiotherapy of appropriately selected meningiomas and metastatic brain tumor beds with gamma knife icon versus volumetric modulated arc therapy

**DOI:** 10.1002/acm2.13100

**Published:** 2020-11-18

**Authors:** Jacob S. Buatti, John M. Buatti, Sridhar Yaddanapudi, Edward C. Pennington, Dongxu Wang, Brandie Gross, Joël J. St‐Aubin, Daniel E. Hyer, Mark C. Smith, Ryan T. Flynn

**Affiliations:** ^1^ Department of Radiation Oncology University of Iowa Hospital and Clinics 200 Hawkins Drive Iowa City IA 52242 USA

**Keywords:** gamma knife, meningioma, stereotactic radiotherapy, tumor bed, volumetric modulated arc therapy

## Abstract

**Purpose:**

To determine if the gamma knife icon (GKI) can provide superior stereotactic radiotherapy (SRT) dose distributions for appropriately selected meningioma and post‐resection brain tumor bed treatments to volumetric modulated arc therapy (VMAT).

**Materials and Methods:**

Appropriately selected targets were not proximal to great vessels, did not have sensitive soft tissue including organs‐at‐risk (OARs) within the planning target volume (PTV), and did not have concave tumors containing excessive normal brain tissue. Four of fourteen candidate meningioma patients and six of six candidate patients with brain tumor cavities were considered for this treatment planning comparison study. PTVs were generated for GKI and VMAT by adding 1 mm and 3 mm margins, respectively, to the GTVs. Identical PTV V_100%_‐values were obtained for the GKI and VMAT plans for each patient. Meningioma and tumor bed prescription doses were 52.7–54.0 in 1.7–1.8 Gy fractions and 25 Gy in 5 Gy fractions, respectively. GKI dose rate was 3.735 Gy/min for 16 mm collimators.

**Results:**

PTV radical dose homogeneity index was 3.03 ± 0.35 for GKI and 1.27 ± 0.19 for VMAT. Normal brain *D*_1%_, *D*_5%_, and *D*_10%_ were lower for GKI than VMAT by 45.8 ± 10.9%, 38.9 ± 11.5%, and 35.4 ± 16.5% respectively. All OARs considered received lower maximum doses for GKI than VMAT. GKI and VMAT treatment times for meningioma plans were 12.1 ± 4.13 min and 6.2 ± 0.32 min, respectively, and, for tumor cavities, were 18.1 ± 5.1 min and 11.0 ± 0.56 min, respectively.

**Conclusions:**

Appropriately selected meningioma and brain tumor bed patients may benefit from GKI‐based SRT due to the decreased normal brain and OAR doses relative to VMAT enabled by smaller margins. Care must be taken in meningioma patient selection for SRT with the GKI, even if they are clinically appropriate for VMAT.

## 1 Introduction

Stereotactic radiosurgery (SRS) using the Leksell Gamma Knife (Elekta AB, Stockholm, Sweden) is a highly accurate radiation delivery modality, with reported end‐to‐end accuracy of under 0.4 mm when the frame‐based approach is used.^1^ The Gamma Knife Icon (GKI), released in 2015, is capable of cone beam computed tomography (CBCT) guided frameless SRS, wherein the patient is immobilized in a face mask and tracked throughout treatment using an infrared camera.^2^, ^3^ In real time, the infrared camera tracks a reflective marker placed on the patient’s nose.

The system has the capability to pause the radiation beam during treatment if the tracked motion is outside of a pre‐defined threshold.^4^ The accuracy of the CBCT‐based GKI positioning system has been reported to be within 0.15 mm of the frame‐based system,^2^, ^5^ enabling 0.5 mm end‐to‐end frameless initial positioning accuracy.

The frameless treatment capability of the GKI is well‐tolerated by patients and enables fractionated stereotactic radiation therapy (SRT) with greater delivery accuracy than conventional SRT delivered with a linear accelerator. Linear accelerator‐based SRT is typically delivered using intensity modulated radiation therapy (IMRT) or volumetric modulated arc therapy (VMAT). Several studies have been published comparing linear accelerator based SRT treatment plans to those of frameless Gamma Knife SRT using a Gamma Knife Perfexion system (pre‐CBCT) and the Extend bite tray system for patient positioning^6^, ^7^, ^8^ and also with the GKI.^9^ In all of these studies, the same margin between gross tumor volume (GTV) and planning target volume (PTV) was used for Gamma Knife as for linear accelerator based SRT, which was reported to be 2–2.5 mm in three of the studies.^6^, ^7^, ^9^ The general findings from these previous studies were that frameless Gamma Knife deliveries provided superior target dose conformity, lower brain dose, and poorer target dose homogeneity than for the linear accelerator‐based approach. Given the accuracy of the CBCT positioning system used by the GKI and intrafraction monitoring throughout treatment, a 1 mm margin for GKI treatments may be justified. For linear accelerator‐based SRT treatments at the authors’ institution and elsewhere, a 3‐mm margin is typically employed.^10^, ^11^ In this work, we conduct an evaluation of the potential benefits of frameless GKI SRT treatments versus linear accelerator based SRT delivered with VMAT using 1 mm and 3 mm GTV‐to‐PTV margins, respectively, for appropriately selected meningioma and post‐resection brain tumor bed treatments.

This is the first study in which the impact of margin reduction for GKI versus linear accelerator based SRT deliveries is accounted for in the treatment planning process at low dose‐per‐fraction values of 1.8 Gy per fraction. Such an approach to treating SRT patients is of interest to enable a reduction in normal brain tissue and organ‐at‐risk (OAR) doses. The approach is also of interest in a center like ours wherein a linear accelerator is replaced with a GKI to enable frame‐based stereotactic radiosurgery, since patients who would be treated with fractionated SRT on the replaced linear accelerator could be instead treated on the GKI, reducing patient load on other linear accelerators at the center.

## 2 Methods and Materials

### 2.A Patient selection

GKI dose heterogeneity was considered in determining candidacy for fractionated GKI therapies. Patients were eliminated from the study based on proximity to great vessels and/or OARs located adjacent to the GTV. Cases in which these risk factors are present may be more safely treated using volumetric modulated arc therapy (VMAT). Where a more homogeneous dose distribution effectively constrains the maximum dose to the sensitive structures within the PTV to values less than or equal to the tolerance dose, which is often 54 Gy in 1.8 Gy fractions.^12^

In two Institutional Review Board approved studies (IRB 20050306 and 201109821, J. M. Buatti, PI), the selection criteria were applied to 14 candidate patients with meningiomas and six candidate patients with post‐operative brain tumor beds. Ten of the 14 patients with meningiomas were rejected for the treatment planning study due to proximity to great vessels, location relative to critical OARs such as the retina within the PTV, tumor location adjacent to sensitive soft tissue near the sagittal sinus, close tumor proximity to many OARs, and/or concave tumor contours that included excessive normal brain tissue. All six patients with post‐operative brain tumor beds were accepted.

### 2.B Images and contouring

Magnetic resonance (MR) images used for planning were acquired using a Siemens Magnetom Tim Trio ST 3.0‐Tesla scanner (Siemens AG, Munich, Germany). Rapid gradient‐echo (MP‐RAGE) images were acquired with 512 × 512 pixels per slice, 0.49 x 0.49 mm^2^ pixels, and 1.0 mm slice thickness. Computed tomography (CT) images were obtained from a Siemens Biograph 40 positron emission tomography/CT scanner. CT image dimensions were 512 × 512 pixels per slice with 0.78 x 0.78 mm^2^ pixels and 1.0 mm slice thickness. GTVs and OARs were contoured on the MR images. MR images were used to define the outer body contour for dose calculation in the GKI plans while the CT images were used for calculating the dose in the VMAT plans.

### 2.C PTV generation and prescriptions

Two PTVs were created for each patient: one for the GKI and one for the VMAT treatment plans. Different margins were used for each system and applied uniformly to the GTV using a research version (5.19.0.d) of the Elekta Monaco treatment planning system (Elekta AB, Stockholm, Sweden). PTVs for VMAT plans were created by adding a uniform 3‐mm margin to the GTVs. The 3‐mm margin is used routinely at the authors’ institution and other centers,^10^, ^11^ and representative of standard margins used for SRT treatments.

A major consideration for GKI planning that is not an issue for VMAT planning due to the physical differences in the paradigms is in determining where to place GKI “shots.” Each shot corresponds to a combination of the eight different ^60^Co beam sectors, wherein each sector contains 24 individual beams all of which are 4 mm, 8 mm, or 16 mm in diameter at the focus point of all beams, or in the off position. For a given shot, each sector can be weighted differently by controlling the amount of time the beams in the sector are turned on. For each shot, the patient may be moved to a different position by the treatment table, which is changing the location of the isocenter inside the patient. Thus, for the GKI, the spatial dose distribution is defined by defining isocenter locations for all shots, and, for each shot, a combination of sectors with the corresponding beam on times and beam diameters. For all VMAT plans, a single isocenter was used and the spatial combination of multi‐leaf collimator positions and beam‐on times for each beam position in the arcs used determine the spatial dose distribution in the patient.

A critical component of GKI planning that is not a part of the VMAT planning in this work is defining where the isocenters can be placed within the patient prior to optimizing the remaining parameters associated with the shot(s) at each isocenter. To accomplish this for the GKI, a planning region at risk volume (PRV) for each OAR was generated by adding a uniform 1 mm margin about the OAR. The PTV was generated by creating a GTV + 1 mm volume for each patient minus all PRVs, and isocenters were only allowed to be within the PTV. This prevented major hotspots from falling within the OARs due to the presence of isocenters within them. The PTV approach was not taken for the VMAT plans since our SRT patients are standardly treated without using the PRV approach since, due to the dose homogeneity achievable with VMAT in combination with the prescription dose levels (Table 1). The PRV approach is unnecessary to achieve acceptable OAR doses and PTV coverage with VMAT.

**Table 1:** Prescription parameters for all patients considered.

Patient	Target	Prescription		
		Dose (Gy)	Fractions	PTV V_100%_
A	Meningioma	52.7	31	97%
B	Meningioma	54.0	30	98%
C	Meningioma	54.0	30	97%
D	Meningioma	54.0	30	96%
F‐K	Brain Tumor Bed	25.0	5	98%

PTV = planning target volume and V_100%_ = percentage of volume receiving 100% of the prescribed dose or higher.

Meningioma and brain tumor bed dose‐volume (dose prescribed to V_100%_, the percentage of the PTV receiving 100% of the prescribed dose or higher) prescriptions ranged from 52.7–54.0 Gy in 1.7 Gy/fx–8 Gy/fx ^13^ and 25 Gy in 5 Gy fractions, respectively, as shown in Table 1. The meningioma prescriptions matched those used clinically for the respective patients.

### 2.D Treatment planning

VMAT treatment plans were created using the Pinnacle treatment planning system version 9.10 (Philips Medical Systems, Fitchburg, WI). The adaptive convolve dose calculation algorithm was used with a 2 mm × 2 mm × 2 mm dose grid. All plans were optimized for treatment on the Elekta Versa HD using 6 MV photon beams. Each plan used a 5 non‐coplanar arc VMAT plan with a 15‐degree collimator rotation for each arc. The beam geometries of the 5 fields were: (1) couch 340°/ gantry 0°‐140°, (2) couch 305°/ gantry 140°‐0°, (3) couch 20°/ gantry 0°‐220°, (4) couch 55°/ gantry 220°‐0°, (5) couch 90°/ gantry 0°‐220°. The linear accelerator’s isocenter was placed approximately at the geometric center of the PTV. The planning objective for each patient was to satisfy the prescription goals of Table 1 without exceeding the OAR tolerance doses. Maximum doses for the brain tumor bed PTVs were limited to under 115% of the prescribed dose (28.75 Gy). The optimization approach for the VMAT plans involved setting a minimum dose objective equal to the prescription dose with a weight of 40–60, a uniform dose objective for 10–20 cGy above the prescription dose with a weight of 80, and a maximum dose objective of 100 cGy above the prescription dose with a weight of 40–60. OAR maximum dose objectives with iteratively‐determined weights that resulted in plans that met the goals (Table 1) were included.

GKI treatment plans were created using Leksell GammaPlan version 11.0.3 (LGP) using the tissue maximum ratio (TMR 10) dose calculation algorithm. A finer resolution was used for the GKI dose calculations than for VMAT due to the much greater dose heterogeneity with the GKI: 0.5 mm × 0.5 mm × 0.5 mm. A dose rate of 3.735 Gy/minute with a 16 mm collimator was used, which was the dose rate within 3 months of the initial ^60^Co source loading. Images and structures were imported into LGP from Monaco to ensure contour consistency between the VMAT and GKI plans. A combination of automated planning tools and manual adjustments were used to meet the dose prescription objectives. Target volumes were filled with spherical shots using the filling program in LGP with uniform filling using shot collimator sizes of 4 mm, 8 mm, 16 mm, determined retrospectively based on the size of the tumor. An initial optimization was performed, with LGP inverse planning settings set to 0.8 coverage, 0.2 selectivity, and less than 0.25 for both beam time and gradient. At least 3,000 iterations of the optimizer were run before termination to ensure minimization of the objective value, and manual optimization was used to achieve the prescription objectives. Shots in GKI treatment that were below the minimum time of 0.1 min were manually deleted. Plans were considered complete when the plan fulfilled the prescription, maximum OAR doses were below tolerance, and a radiation oncologist approved them.

### 2.F Treatment plan evaluation

GKI and VMAT plans were compared using the radical dose homogeneity index (rHI), maximum and mean OAR doses, and normal brain tissue *D*_1%_, *D*_5%_, and *D*_10%_, where *D_x_*_%_ is the minimum dose, in Gy, received by the hottest *x*% of the normal brain. The rHI metric is the ratio of the maximum to the minimum PTV doses. Normal brain tissue was defined as the brain minus the GTV, and was generated using Velocity software, version 3.2.1 (Varian Medical Systems, Palo Alto, CA, USA).

## 3 Results

**Fig 1:**
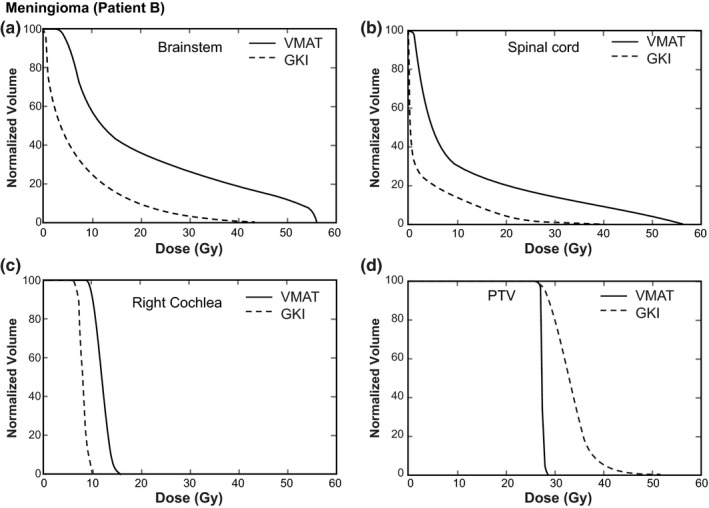
Example dose volume histograms for meningioma Patient B showing (a) brainstem, (b) spinal cord, (c), right cochlea, and (d) the planning target volume (PTV).

**Fig 2:**
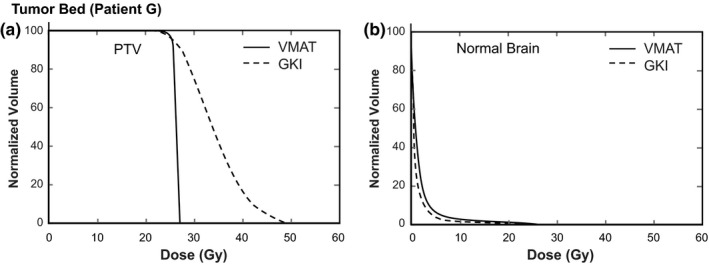
Example dose volume histograms for tumor cavity patient G showing (a) planning target volume (PTV) and (b) normal brain. There were no other organs at risk close enough to the PTV to be considered.

For all patients, the V_100%_ prescription goals were met for both the VMAT and GKI plans (Table 2). The rHI, normal brain *D*_1%_, *D*_5%_, and *D*_10%_ values, and treatment times for GKI (as predicted by GammaPlan) are listed in Table 2. PTV dose homogeneity was 3.03 ± 0.35 for GKI and 1.27 ± 0.19 (mean ± standard deviation) for VMAT. Over all meningioma and brain tumor cavity patients, rHI was 136 ± 27% (mean ± 1 standard deviation) greater in GKI plans than VMAT plans (Table 2). As shown in Table 3, all OARs received a lower maximum dose from GKI than VMAT, and ten of the eleven OARs from the 4 meningioma cases received a lower mean dose from GKI than VMAT. Over all meningioma and brain tumor cavity patients, the normal brain *D*_1%_, *D*_5%_, and *D*_10%_ were 45.8 ± 10.9%, 38.9 ± 11.5%, and 35.4 ± 16.5% lower for GKI than for VMAT treatments. Dose volume histograms for a meningioma patient and a tumor cavity patient are shown in Fig. 1 and Fig. 2, respectively, which indicate the decreased OAR doses and increased PTV dose heterogeneity for GKI versus VMAT plans.

**Table 2:** Treatment planning results for all patients considered.

Patient	Type	PTV V_100%_ (%)		rHI		Normal Brain Dose (Gy)						Treatment Time (min) for GKI
						GKI			VMAT			
		GKI	VMAT	GKI	VMAT	*D*_1%_	*D_5_*_%_	*D*_10%_	*D*_1%_	*D*_5%_	*D*_10%_	
A	MG	97	97	3.14	1.06	15.6	6.3	4.1	38.2	12.5	8.1	9.2
B	MG	98	98	2.62	1.09	16.3	7.1	4.8	39.9	13.5	8.1	13.7
C	MG	97	97	3.78	1.79	39.6	22.1	15.7	55.4	42.9	26.8	17.1
D	MG	96	96	2.78	1.26	16.8	6.9	4.6	43.8	16.3	10.8	8.2
F	TB	98	98	3.25	1.25	12.2	4.8	3.0	22.8	6.9	4.1	18.6
G	TB	98	98	2.60	1.19	12.6	4.5	2.6	21.8	6.5	3.8	11.9
H	TB	98	98	3.07	1.23	6.2	6.2	3.5	25.0	9.5	5.4	21.2
I	TB	98	98	3.13	1.47	15.4	6.1	3.9	24.6	9.2	5.3	21.2
J	TB	98	98	2.86	1.26	15.8	7.8	5.1	25.5	9.3	4.8	23.9
K	TB	98	98	2.75	1.24	9.5	3.4	2.1	18.6	5.1	3.8	11.9

MG, meningioma; TB, brain tumor bed; PTV, planning target volume; V_100%_, percentage volume receiving 100% of prescribed dose or higher; GKI, Gamma Knife Icon; VMAT, volumetric modulated arc therapy; *D*_x%_, minimum dose to hottest *x*% of volume; rHI = radical dose homogeneity index.

**Table 3:** Maximum point doses and mean doses to relevant organs at risk in meningioma patients.

Patient	Organ at risk	Maximum dose (Gy)		Mean dose (Gy)	
		GKI	VMAT	GKI	VMAT
A	Optic chiasm	53.5	53.9	24.3	49.4
A	Left optic nerve	52.8	53.6	7.8	27.9
A	Right optic nerve	53.4	54.3	9.2	15.4
B	Brainstem	54.4	56.6	7.3	20.5
B	Right cochlea	10.7	15.9	8.2	12.0
B	Spinal cord	46.7	55.7	3.8	12.3
C	Brainstem	54.7	57.0	24.4	45.4
C	Optic chiasm	34.9	51.6	22.3	19.8
C	Left cochlea	54.0	55.1	47.7	54.6
D	Optic chiasm	24.9	53.5	11.0	15.5
D	Left optic nerve	53.5	55.0	19.3	23.9

GKI, gamma knife icon; VMAT, volumetric modulated arc therapy.

GKI beam‐on times reported by LGP were 12.1 ± 4.13 min for meningioma plans and 18.1 ± 5.1 min for tumor cavities (Table 2). VMAT beam‐on times are not provided by the Pinnacle treatment planning system, and, based on our oncology information system (Mosaiq), average beam‐on times are 6.2 ± 0.32 min for 1.8 Gy fractions and 11.0 ± 0.56 min for 5 Gy fractions.

## 4 Discussion

The results of this work indicate that SRT delivered with a GKI can be dosimetrically superior to that delivered with VMAT for selected meningioma patients and brain tumor bed patients, due specifically to OAR dose and normal brain dose reduction through GTV‐to‐PTV margin reduction. The increased PTV dose heterogeneity of the GKI poses a danger for patients with great vessels in close proximity to the GTV, and such patients were ruled out as GKI candidates for this study. In properly selected patients, high‐dose regions within the GTV owing to increased dose heterogeneity pose no expected increase in complication risk and may improve tumor control. In all GKI treatments, the average dose to the GTV is increased and the dose to the surrounding normal tissue is lower and/or below tolerance levels.

It has been shown in previous studies that frameless Gamma Knife deliveries can provide as good as or better PTV dose conformity to linear accelerator based intensity modulated radiation therapy.^6^, ^7^, ^8^, ^9^ However, the superior accuracy of the GKI suggests that a smaller margin is appropriate to create PTVs for the GKI than for linear accelerator‐based approaches. The GKI at our institution has been shown in an in‐house phantom study (results not published) to have a frameless end‐to‐end positioning accuracy of 0.4 mm, which accounts for mechanical focal point accuracy, table positioning uncertainties, cone‐beam CT accuracy and image registration accuracy. This accuracy is supported by published results.^5^ Despite system inaccuracies, margins have not traditionally been used for single fraction Gamma Knife treatments.^14^ Since increased inaccuracy and movement is possible during frameless GKI treatment, a conservative 1 mm margin was used in the GKI treatment plans.

Given the increased spatial accuracy of the GKI and the heterogeneity of GKI dose distributions, it was determined to be inappropriate to use the same GTV‐to‐PTV margins and the same OAR‐prioritization approach for both GKI and VMAT. We found that generating the GKI PTVs that accounted for the increased accuracy of GKI (1 mm rather than 3 mm margins), and on which the same V_100%_ could be achieved as for VMAT, necessitated a PRV approach to avoid overdosing OARs adjacent to the GTV. This is because margins added to the GTV often create a volume that intersects with normal tissue and OARs in the surroundings of the GTV. For OARs that are closer than 1 mm to the GTV, it is possible that the associated PRV margin will overlap with the GTV. In that case, whether or not the patient should be excluded from consideration for GKI treatment would be subject to the clinical judgment of the physician. GKI treatments are normalized to a maximum dose point and prescribed to a percentage of the maximum dose, typically 50%. Due to GKI dose heterogeneity inherent to the modality and prescriptions being equal to or greater than OAR tolerance limits, OAR doses may easily exceed their tolerances if the PTV overlaps the OAR and is not specifically accounted for in the planning process. The planning technique used in the current work, in which GKI PTVs were generated by subtraction of PRVs from GTV + 1 mm volumes, is designed to avoid placing hot spots inside OARs and is indicative of the dramatically higher dose heterogeneity that the GKI delivers relative to VMAT.

**Fig 3:**
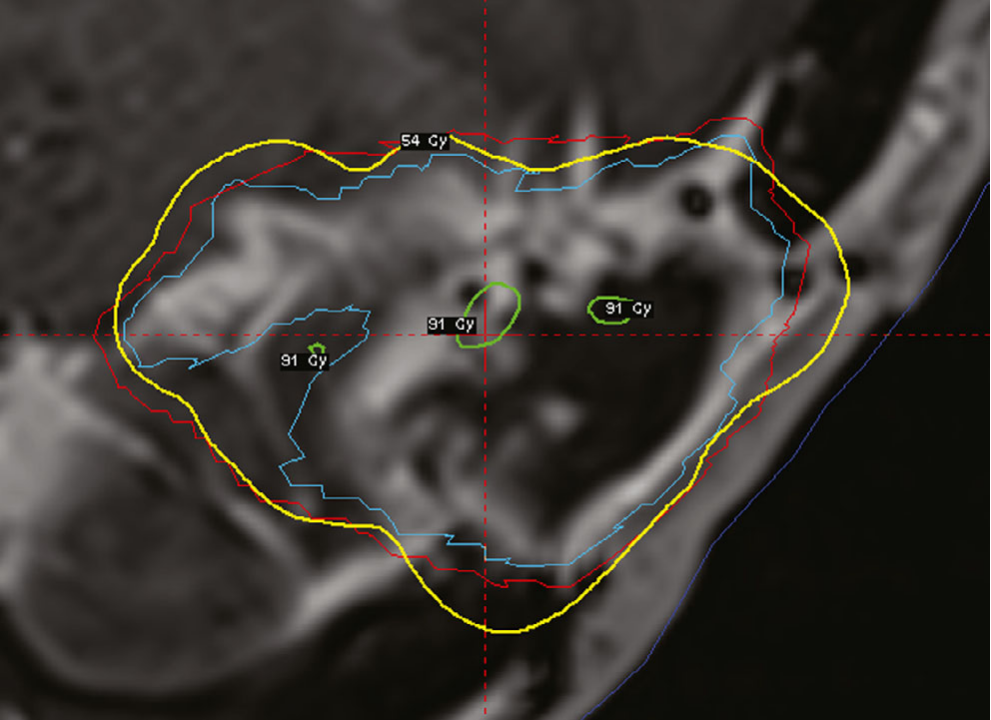
GKI plan for a meningioma with a complex GTV (blue) contour with a concavity of normal brain that was included in the PTV (red), resulting in a hot spot within normal brain tissue that could not be removed without unacceptably compromising PTV V_100%_. The yellow line is the 54 Gy isodose line.

In some GKI plans, the maximum normal brain dose between the GTV and PTV was increased substantially above that of VMAT due to normal brain concavities within the GTV that could not be spared without unacceptably lowering dose within the PTV. VMAT plans, with optimization constraints that enforce dose homogeneity (and thus reduce hot spots within the target) can avoid this issue, whereas the location of hot spots needs to be considered explicitly during the GKI planning process. However, due to the complexity of placing shots within the target for GKI, it may not always be possible to eliminate hot spots between the GTV and PTV completely. An example of this is shown in Fig. 3, a patient that was excluded from consideration for GKI, where a normal brain tissue concavity is partially surrounded by GTV (blue) within the PTV (red), resulting in a small volume of normal brain tissue receiving a high dose of 91 Gy to ensure the PTV dose‐volume goal is achieved. The normal brain *D*_1%_, *D*_5%_, and *D*_10%_ for the patient shown in Fig. 3 were 30%, 87% and 170% higher, respectively, in the GKI plan than the VMAT plan, due to target contour concavity, hence the exclusion.

When treating with VMAT, the dose homogeneity achieved within the margin between the GTV and PTV can maintain the dose to the normal brain adjacent to the GTV at or below the GTV prescription dose, which is known from clinical experience to be acceptably tolerated by patients. The lack of dose homogeneity within the margin that is characteristic of the GKI, even with a reduced margin relative to the linear accelerator‐based approach, can drive the hot spots above levels that may be clinically acceptable. This issue has been reported previously in the context of 5‐fraction SRT.^8^, ^14^ In the current work 30‐31 fraction SRT for meningiomas was also considered. It was found that a subset of those patients (4 of 14) would still be candidates for GKI‐based SRT, and those patients would potentially benefit from substantially lower normal brain *D*_1%_, *D*_5%_, and *D*_10%_‐values than VMAT without compromising OAR doses. Care would need to be taken to exclude patients for whom dramatically higher normal brain hot spots, close OARs, or the close proximity of great vessels adjacent to the GTV would result in added risk beyond VMAT due to the inherent dose inhomogeneity of the GKI.

## 5 Conclusion

Appropriately selected meningioma patients and brain tumor bed patients may benefit from GKI‐based SRT due to the decreased normal brain and OAR doses relative to VMAT enabled by smaller margins. Due to dose the dose heterogeneity of the GKI and despite the potential for reduced OAR and normal brain sparing for some patients, care must be taken in meningioma patient selection for SRT with the GKI even if they are clinically appropriate for VMAT.

## Author contribution statement

**Jacob S. Buatti** conducted treatment Gamma Knife treatment planning (lead); formal analysis (lead); writing original draft (lead); review and editing (equal). **John M. Buatti contributed to** conceptualization (lead); contouring for treatment planning (lead); reviewed treatment plans (lead); review and editing (equal). **Sridhar Yaddanapudi contributed to** treatment planning (supporting); formal analysis (supporting); software management (equal). **Edward C. Pennington contributed to** software management (equal); review and editing (equal). **Dongxu Wang contributed to** software management (equal); review and editing (equal). **Brandie Gross contributed to** treatment planning for VMAT plans. **Joël J. St‐Aubin and Daniel E. Hyer contributed to** review and editing (equal); treatment planning for Gamma Knife (supporting). **Mark C. Smith contributed to** contouring for treatment planning (supporting); review and editing (equal). **Ryan T. Flynn contributed to** writing original draft (supporting); review and editing (equal); project management (lead); formal analysis (supporting); methodology (lead); supervised first author (lead).

## References

[1] Brezovich IA , Wu X , Duan J , et al. End‐to‐end test of spatial accuracy in gamma knife treatments for trigeminal neuralgia. Med Phys. 2014;41:111703. 25370617 10.1118/1.4896819

[2] Zeverino M , Jaccard M , Patin D , et al. Commissioning of the leksell gamma knife((r)) icon. Med Phys. 2017;44:355–363. 28133748 10.1002/mp.12052

[3] Stieler F , Wenz F , Schweizer B , et al. Validation of frame‐based positioning accuracy with cone‐beam computed tomography in gamma knife icon radiosurgery. Phys Med. 2018;52:93–97. 30139616 10.1016/j.ejmp.2018.06.640

[4] Wright G , Harrold N , Hatfield P , Bownes P . Validity of the use of nose tip motion as a surrogate for intracranial motion in mask‐fixated frameless gamma knife((r)) icon therapy. J Radiosurg SBRT. 2017;4:289–301. 29296453 PMC5658824

[5] AlDahlawi I , Prasad D , Podgorsak MB . Evaluation of stability of stereotactic space defined by cone‐beam ct for the leksell gamma knife icon. J Appl Clin Med Phys. 2017;18:67–72. 28419781 10.1002/acm2.12073 PMC5689865

[6] Nakazawa H , Mori Y , Komori M , et al. Simulational study of a dosimetric comparison between a gamma knife treatment plan and an intensity‐modulated radiotherapy plan for skull base tumors. J Radiat Res. 2014;55:518–526. 24351459 10.1093/jrr/rrt136 PMC4014159

[7] Huss M , Barsoum P , Dodoo E , et al. Fractionated srt using vmat and gamma knife for brain metastases and gliomas–a planning study. J Appl Clin Med Phys. 2015;16:3–16. 10.1120/jacmp.v16i6.5255 PMC5691017 26699547

[8] Dong P , Pérez‐Andújar A , Pinnaduwage D , et al. Dosimetric characterization of hypofractionated gamma knife radiosurgery of large or complex brain tumors versus linear accelerator‐based treatments. J Neurosurg. 2016;125:97–103. 27903198 10.3171/2016.7.GKS16881

[9] Han EY , Wang H , Luo D , Li J , Wang X . Dosimetric comparison of fractionated radiosurgery plans using frameless gamma knife icon and cyberknife systems with linear accelerator‐based radiosurgery plans for multiple large brain metastases. J Neurosurg. 2019;1–7. 10.3171/2019.1.JNS182769 30952125

[10] El Shafie RA , Czech M , Kessel KA , et al. Clinical outcome after particle therapy for meningiomas of the skull base: toxicity and local control in patients treated with active rasterscanning. Radiat Oncol. 2018;13:54. 29587795 10.1186/s13014-018-1002-5 PMC5870393

[11] Onodera S , Aoyama H , Katoh N , et al. Long‐term outcomes of fractionated stereotactic radiotherapy for intracranial skull base benign meningiomas in single institution. Jpn J Clin Oncol. 2011;41:462–468. 21177777 10.1093/jjco/hyq231

[12] Marks LB , Yorke ED , Jackson A , et al. Use of normal tissue complication probability models in the clinic. Int J Radiat Oncol Biol Phys. 2010;76:S10–19. 20171502 10.1016/j.ijrobp.2009.07.1754 PMC4041542

[13] Condra KS , Buatti JM , Mendenhall WM , et al. Benign meningiomas: Primary treatment selection affects survival. Int J Radiat Oncol Biol Phys. 1997;39:427–436. 9308947 10.1016/s0360-3016(97)00317-9

[14] Ma L , Sahgal A , Larson DA , et al. Impact of millimeter‐level margins on peripheral normal brain sparing for gamma knife radiosurgery. Int J Radiat Oncol Biol Phys. 2014;89:206–213. 24725703 10.1016/j.ijrobp.2014.01.011

